# Pathogen landscape and synergistic interactions in pediatric ARIs: implications for broad-spectrum surveillance via targeted NGS

**DOI:** 10.1128/spectrum.02275-25

**Published:** 2025-12-30

**Authors:** Jingjiao Zhou, Ting Li, Shenzhen Yuan, Yi Wang, Alan Zhao, Yifan He, Xiaoyun Zhan, Bei Wu, Zhiping Jia, Yi Liu, Jun Li, Han Yang, Jun Zhou, Zhongyi Wang, Juan Huang

**Affiliations:** 1 Department of Biology and Genetics, The College of Life Sciences and Health, Wuhan University of Science and Technology 47900, Hubei, China; 2 Department of Paediatrics, Maternal and Child Health Hospital of Hubei Province, Tongji Medical College, Huazhong University of Science and Technology 12443, Hubei, China; 3 Institute of Biology and Medicine, The College of Life Sciences and Health, Wuhan University of Science and Technology, Wuhan, China; University of Nebraska-Lincoln, Lincoln, Nebraska, USA

**Keywords:** tNGS, respiratory pathogen, co-infection, blood biochemistry indicator, clinical characteristics

## Abstract

Acute respiratory infections (ARIs) in children present complex interactions between respiratory pathogens and host, contributing to high morbidity, hospitalization, and mortality. In this cross-sectional study, 2,044 pediatric in-patient samples were tested for 198 pathogens using targeted next-generation sequencing (tNGS). Pathogen spectrum and co-infection pattern were analyzed alongside blood-test results and clinical data. Pediatric ARIs presented staggered viral epidemics and age-specific infection patterns from 2022 to 2023. Viral infections were predominant in children under 3 years (67.5%). Specific viral-bacterial co-infections were associated with disease severity, including human metapneumovirus (HMPV)-*Streptococcus pneumoniae* (OR = 3.172, 95% CI: 1.257–8.009), human parainfluenza virus (HPIV)-*Fusobacterium nucleatum* (3.016, 1.051–8.653), and HPIV-*S. pneumoniae* (2.825, 1.007–7.927). Subnormal levels of prealbumin and creatinine served as biomarkers for homeostasis disruption in viral-bacterial co-infections. From June to December 2023, *Mycoplasma pneumoniae* (MP) became the dominant pathogen in preschool (31.3%) and school-age (45.3%) children. High MP loads were correlated with elevated eosinophil, IL-4, and IL-6 and decreased IgG and IL-2, linking to airway hyperreactivity and inflammation. At low MP loads, patients co-detected with human herpesvirus (HHV) (50.0%), or *Haemophilus influenzae* (40.0%) had a high proportion of severe cases. This study delineated the complex pathogen landscape of pediatric ARIs and highlighted the major role of viral-bacterial and MP-bacterial co-infections. Monitoring these pathogens through tNGS can aid in precise diagnosis and targeted treatment to improve clinical outcomes in children.

**IMPORTANCE:**

Moving beyond the outdated “one germ, one disease” model, this study characterizes the etiological and epidemiological landscape and highlights co-infection patterns of virus-bacterium or MP-bacterium in 2044 pediatric respiratory in-patient children across three epidemic phases. Specific pathogen combinations are associated with clinical severity, as well as host immune and metabolic profiles. Our findings underscore the necessity of detecting pathogen-pathogen and host-pathogen dynamics, rather than individual pathogens, thereby informing precision diagnostics and targeted intervention strategies.

## INTRODUCTION

Acute respiratory infections (ARIs) remain a significant global health challenge in pediatric populations, contributing to high morbidity, hospitalization rates, and mortality, particularly in resource-limited settings. Lower respiratory infection is the leading cause of death in children under 5 years of age, resulting in over 740,000 deaths and accounting for 14% of total deaths in 2019 (1, 2).

The clinical complexity of pediatric ARIs is further compounded by the intricate polymicrobial landscape, unusual seasonal fluctuations, and emerging antimicrobial resistance. These altered patterns have posed considerable challenges to clinical diagnosis and treatment in pediatric ARI cases, which typically present with more severe clinical manifestations and acute symptom onset (3–5). Therefore, enhanced pathogen surveillance, refined etiological characterization, and targeted therapeutic approaches in pediatric respiratory medicine are an urgent need.

Traditional culture-based pathogen detection methods often fail to meet the demands of clinical diagnosis, driving the development of next-generation sequencing (NGS)-based pathogen identification. Targeted next-generation sequencing (tNGS), which integrates ultra-multiplex PCR with high-throughput sequencing, has proven clinical utility in practical applications with high sensitivity and rapid reporting. Compared to metagenomics next-generation sequencing, tNGS exhibits strengths in detecting pathogen virulence factors and antimicrobial resistance genes, while maintaining a well-defined detection spectrum and reduced operational costs (6–9).

Herein, we collected 2,044 samples of pediatric ARI cases from the Maternal and Child Health Hospital of Hubei Province. Samples were divided into three periods: phase I (June to December 2022), phase II (January to May 2023), and phase III (June to December 2023). All 2,044 samples underwent tNGS testing and analyses to characterize the pathogen spectrum, co-infection patterns, and prevalence trends. In phase II, viruses co-infected with bacteria/fungi emerged as predominant patterns of pediatric ARIs, while the resurgence of MP co-infected with bacteria/fungi was observed in phase III.

Integrating tNGS results with hematological and biochemical data revealed the load-related characteristics of MP infections, and the different clinical characteristics of viral mono-infections or viral-bacterial co-infections. Our findings suggest certain pathogen combination patterns and host immune/metabolic status drive clinical trajectories. The study advocates pathogen landscape monitoring through targeted NGS, providing insights into clinical diagnosis and surveillance in pediatric ARIs.

## MATERIALS AND METHODS

### Study design and patient information

This cross-sectional study included pediatric ARI in-patients from the Maternal and Child Health Hospital of Hubei Province, Tongji Medical College, Huazhong University of Science and Technology, from 1 June 2022 to 31 December 2023. Inclusion criteria included (i) diagnosis of respiratory infections; (ii) undergoing tNGS pathogen detection; (iii) complete patient information and clinical data availability; and (iv) informed consent for study participation. Exclusion criteria included (i) refusal of tNGS detection; (ii) oropharyngeal swab samples failed to meet tNGS quality standards; and (iii) incomplete clinical data.

The collected samples were categorized into three phases: phase I, June to December 2022 (summer–autumn, under dynamic zero-COVID policies); phase II, January to May 2023 (winter–spring, post-NPIs); phase III, June to December 2023 (summer–autumn, post-NPIs).

The severity of each pediatric patient was assessed by clinicians based on clinical symptoms, physical signs, radiographic features, and laboratory test results. Assessment criteria were referenced from the following authoritative guidelines: Guidelines for the diagnosis and treatment of childhood *Mycoplasma pneumoniae* pneumonia (2023), Guidelines for the diagnosis and treatment of community-acquired pneumonia in children (2019), and Expert consensus on clinical early warning and early decision of severe pneumonia in children. Severe ARI was defined as cases meeting one or more of the following criteria:

1. Persistent fever lasting more than 7 days.
2. Wheezing, tachypnea (respiratory rate ≥70/min in infants or ≥50/min in children older than 1 year), dyspnea, chest pain, hemoptysis, or altered consciousness.
3. Resting pulse oxygen saturation (SpO₂) ≤0.92 while breathing room air.
4. Radiologic evidence of (i) homogeneous consolidation involving ≥2/3 of a single lung lobe, or (ii) diffuse involvement of a single lung or ≥4/5 lobes bilaterally with bronchiolitis features, possibly accompanied by atelectasis.
5. Progressive worsening of clinical symptoms, with radiological evidence showing more than 50% progression of lesions within 24–48 h.
6. Presence of extrapulmonary complications.
7. Significant elevation of any of the following laboratory markers: C-reactive protein, lactate dehydrogenase, D-dimer, procalcitonin, or lactate.

### Specimen information

Fasting venous blood was collected the morning after admission for laboratory tests, including complete blood count, blood chemistry, cytokine panel, etc. Oropharyngeal swab specimens were collected before treatment: children rinsed their mouths with saline, followed by gentle swabbing (2 cm into the oropharyngeal isthmus using a sterile swab), then stored in a specialized sampling tube.

### Targeted next-generation sequencing

Samples were transferred on dry ice to KingMed Diagnostics Co., Ltd. for tNGS testing within 24 h. This assay encompasses 198 pathogens. Following the manufacturer’s protocol, samples were preprocessed, and libraries were prepared using the Respiratory 100 Premixed Reagent Kit (KS608-100HX, KingCreate, China).

Constructed library quality and quantity were evaluated by Qsep100 Biofragment Analyzer (Bioptic, China) and Qubit 4.0 Fluorometer (Thermo Scientific, USA). Library fragments were approximately 250–350 bp, with a minimum concentration of 0.5 ng/µL. The pooled library concentration was reassessed and diluted to 1 nmol/L. Subsequently, 5 µL of the library was mixed with 5 µL NaOH (0.1 mol/L). After brief vortexing and centrifugation, the library was incubated at room temperature for 5 min. Finally, the diluted and denatured library was sequenced using the universal sequencing kit (KS107-CXR, KingCreate, China) on the KM MiniSeqDx-CN sequencing platform (KingCreate, China).

### tNGS data analysis and pathogen identification

KingCreate bioinformatics software (v3.7.2) was employed to filter the sequencing data and align it with reference genomes to interpret the pathogen detection results. The reported pathogen read counts were normalized as the number of pathogen-specific reads per 100 kilobases of total raw sequencing reads in each sample. Semi-quantitative classification was conducted according to the algorithm embedded in the tNGS analysis platform (KingCreate v3.7.2, KingMed Diagnostics), based on the amplification efficiency relationship between the pathogen and the internal reference control. Data quality requirements include (i) Q30 ≥75%, (ii) a minimum of 50,000 raw reads, and (iii) normalized reads count ≥200 for internal controls or ≥3,000 for targeted pathogen regions. For assessment, pathogens with at least one target having normalized read counts of ≥20 were considered positive; otherwise, negative. Further analyses excluded pathogens with reported sequence counts <100 or estimated concentration <10^3^ copies/mL.

### Laboratory procedures

Fasting venous blood was collected and employed to classify and count the blood cells using an Auto hematology analyzer BC 6800 (Mindray, China). Serum was obtained and conducted biochemical tests, including panels of liver and kidney function indicators, myocardial enzymes, and electrolytes using an automatic biochemical analyzer (AU5800, Beckman Coulter, USA). Immune function assays included complement C3, complement C4, immunoglobulin M (IgM), immunoglobulin A (IgA), and immunoglobulin G (IgG). Cytokines, including IL-2, IL-4, IL-6, IL-10, TNF-α, and IFN-γ, were detected using the Cytokine Multiplex Detection Kit (CellGer, China) on FACSCanto Flow Cytometry Systems (BD, USA).

### Data collection and management

Data were collected by reviewing medical records, which included demographic information, timeline of symptom onset, hospital admission, clinical presentations, laboratory testing results, tNGS and other microbiological test results, radiographic findings, medication regimens, and clinical outcomes. These data were systematically entered into a standardized database by trained clinicians. Researchers thoroughly reviewed the individual and clinical data for each enrolled ARI patient.

### Statistical analysis

Descriptive statistics included categorical variables expressed as frequencies (proportions) and continuous variables represented as medians with interquartile ranges (IQRs). Cases with missing values were excluded from that particular analysis. Comparisons were performed using the chi-squared (χ²) test.

The positive detection rate of pathogens was defined as the ratio of the number of detected cases of a given pathogen to the total number of samples tested. The co-infection rate was defined as the number of samples with both given pathogens detected, divided by the total tested cases in each group. Multivariate logistic regression was performed to examine the potential association between severe ARIs and the co-infections of respiratory pathogens, with the odds ratio (OR) and 95% CI estimated using maximum likelihood methods. The Benjamini–Hochberg procedure was applied to control the false discovery rate and reduce the likelihood of false-positive findings. Pearson correlation analysis was used to determine the correlation coefficients between clinical indicators and MP concentration.

All statistical analyses were conducted in R (version 4.1.3), with results visualized using the ggplot2, ggpubr, and simplevis packages. *P* value <0.05 was considered statistically significant.

## RESULTS

### Patient demographics and tNGS sample information included in the study

This study enrolled 2,044 pediatric in-patients with ARIs at the Maternal and Child Health Hospital of Hubei Province from June 2022 to December 2023. We categorized the research period into three intervals: phase I (June to December 2022), phase II (January to May 2023), and phase III (June to December 2023).

A total of 2,044 throat swab samples were collected and tested for 198 pathogens with tNGS. Pathogens with a concentration <10^3^ were filtered out during quality control. Among all samples, 101 (4.9%) had no pathogen detected, while 1,943 (95.1%) tested positive for one or more pathogens. The characteristics of the patients are presented in Table S1. During phases I and II, most patients were aged 1–3 years, while in phase III, the proportion of patients over 4 years old elevated, with a median age of 4 [IQR (2, 7)].

### The spectrum of respiratory pathogens across three defined phases

**Fig 1:**
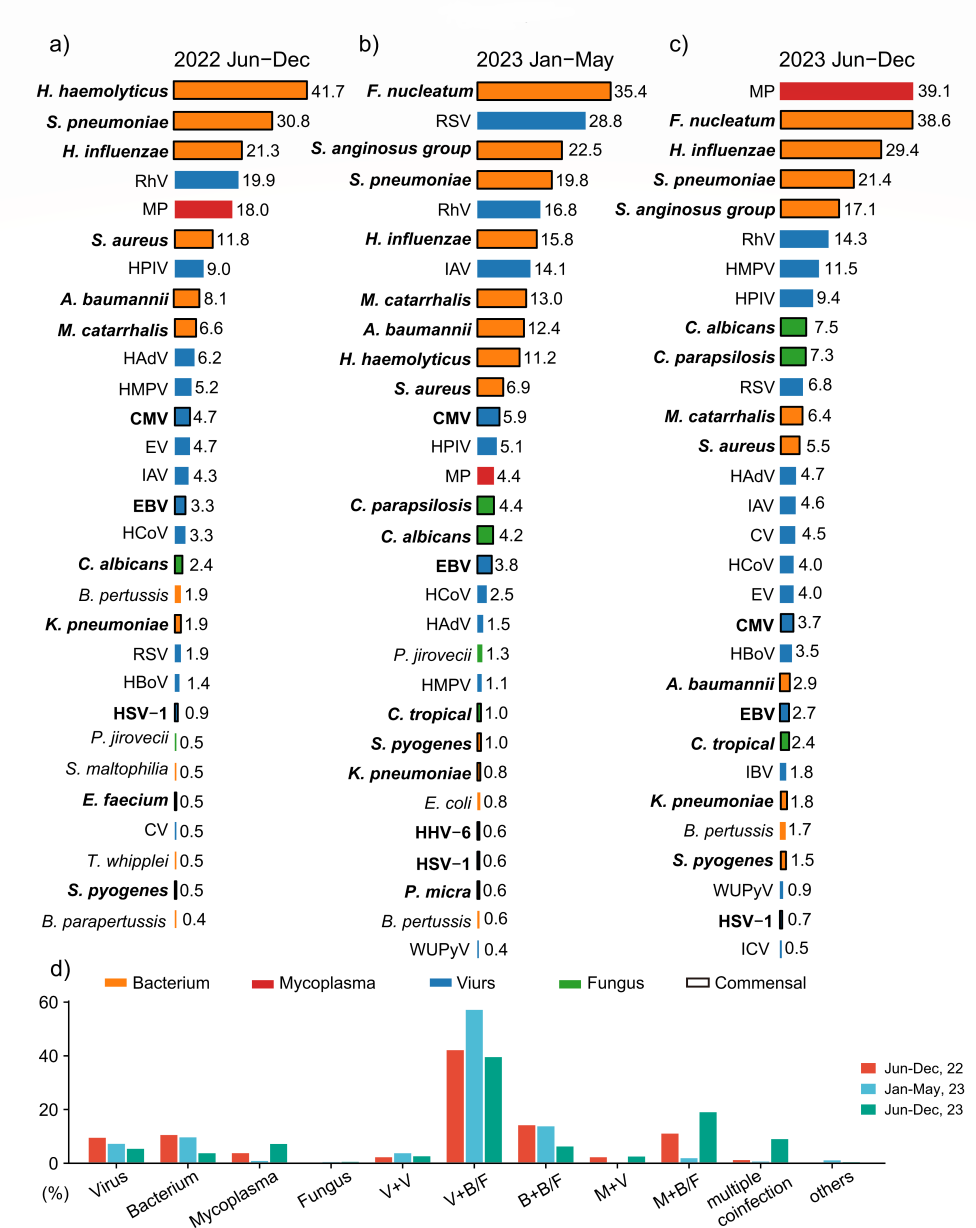
Positive rate of respiratory pathogens across the three defined phases. (**a–c**) Children hospitalized for respiratory infections at Maternal and Child Health Hospital of Hubei Province from June to December 2022 (**a**), January to May 2023 (**b**), or June to December 2023 (**c**) underwent tNGS testing. Bar plots present the positive rate of each pathogen during three phases. The color of the bars corresponds to different pathogen classifications, and the bordered bars indicate respiratory commensal microorganisms. (**d**) Infectious patterns across different phases from June 2022 to December 2023. V, virus; B, bacterium; M, mycoplasma; F, fungus; CMV, cytomegalovirus; CV, coxsackievirus; EBV, Epstein-Barr virus; EV, enterovirus; HAdV, human adenovirus; HMPV, human metapneumovirus; HBoV, human bocavirus; HHV-6, human herpesvirus 6; HCoV, human coronavirus; HSV-1, herpes simplex virus type 1; HPIV, human parainfluenza virus; IAV, influenza A virus; IBV, influenza B virus; ICV, influenza C virus; MP, mycoplasma pneumoniae; RhV, rhinovirus; RSV, respiratory syncytial virus; WUPyV, WU polyomavirus.

Figure 1 displays the top 30 detected pathogens and their positivity rates in each phase, respectively. In phase I, bacterial pathogens were the most prevalent, with *Haemophilus haemolyticus* being the most common (41.7% positive of the total detections in phase I), followed by *Streptococcus pneumoniae* (30.8%) and *Haemophilus influenzae* (21.3%). *Mycoplasma pneumoniae* (MP) was the fifth most frequently detected pathogen (18.0%). The most common viruses detected were human rhinovirus (RhV) (19.9%), human parainfluenza virus (HPIV) (9.0%), and human adenovirus (HAdV) (6.2%). Among the fungi detected, *Candida albicans* was the most prevalent (2.4%), followed by *Pneumocystis jirovecii* (0.5%) (Fig. 1a).

The most common bacteria were *Fusobacterium nucleatum* (35.4%) in phase II, followed by the *Streptococcus anginosus* group (22.5%) and *S. pneumoniae* (19.8%). The most frequently detected viruses were human respiratory syncytial virus (RSV) (with a high prevalence of 28.8%), RhV (16.8%), and influenza A virus (IAV) (14.1%). *Candida parapsilosis* (4.4%) and *C. albicans* (4.2%) were relatively common fungi (Fig. 1b).

In phase III, MP was the most prevalent pathogen (39.1%), followed by the respiratory commensals *F. nucleatum* (38.6%), *H. influenzae* (29.4%), and *S. pneumoniae* (21.4%). Compared to the same period last year (phase I), the positive rate of RhV (14.3%) decreased by 5%, while human metapneumovirus (HMPV) (11.5%) increased by 6.3%. HPIV maintained relatively high rates (9.7%). The most common fungi were *C. albicans* (7.5%) and *C. parapsilosis* (7.3%). Notably, the positive rate of fungal detection in phase III was higher than in phases I and II, highlighting the need for vigilance against fungal infections in immunocompromised individuals (Fig. 1c).

Across the three phases, the incidence of single-pathogen infections was relatively low. The co-positive rates of viruses with bacteria or fungi were the highest, with phase I at 38.9% (82/211), phase II reaching 53.5% (281/525), and phase III at 38.8% (508/1308). MP mono-infection accounted for 7.3% (96/1308), MP-virus co-infection for 2.8% (36/1308), and the highest rate was MP-bacteria/fungi co-infection, reaching 18.9% (247/1308). We also noted “multiple co-infection” (defined as the presence of >2 different classified pathogens) in 1.4% (3/211) of cases in phase I, 0.9% (5/525) in phase II, and 9.1% (119/1308) in phase III (Fig. 1d).

### The prevalence trends of viruses among pediatric patients in Wuhan, June 2022 to December 2023

By the end of 2022, following the comprehensive removal of NPIs in China mainland, Wuhan experienced an intense outbreak of the Omicron variant of SARS-CoV-2, which spanned from mid-December 2022 to late January 2023 (10). During this period, CMV was positively rated for 18.0% among pediatric patients in January, coinciding with the Omicron wave. From March to October 2023, EBV (1.8%–6.1%) and CMV (2.4%–8.2%) remained detectable in pediatric patients (Fig. S1A).

The typical winter flu season was disrupted, with no cases detected during the Omicron epidemic. The IAV wave reached a peak of 34.1% (47/138) in March 2023 and persisted until June (Fig. S1B). The positive detections of RhV (39.5%), RSV (56.3%), and HPIV (28.4%) peaked in October 2022, April 2023, and June 2023, respectively (Fig. S1C).

### The prevalence trends of MP, respiratory bacteria, and fungi among pediatric patients in Wuhan, June 2022 to December 2023

The global incidence of MP was notably reduced by COVID-19 and the NPIs (0.7%–1.69%) (11). The positivity rate for MP began to rise gradually from April 2023, reaching a peak in October at 60.4%, which was 25.1% higher than the subscale peak observed in September 2022 (Fig. S2A).

*H. haemolyticus* (46.2%–50.0%), *H. influenzae* (12.8%–30%), and *Moraxella catarrhalis* (10.3%–20.0%) were frequently detected in children from January to February 2023 (Fig. S2B). In this study, the tNGS panel encompassed 24 fungal types, of which only five species were detected, with *C. albicans* and *C. parapsilosis* being the most prevalent. The positive rates of *C. albicans* (3.0%–10.5%) and *C. parapsilosis* (3.6%–9.6%) had an upward fluctuation from March to December 2023 (Fig. S2C).

### Virus predominance in children under 3 years old, and RhV-*F. nucleatum*, RhV-*H. influenzae*, and HMPV-*S. pneumoniae* are the most common co-infections

**Fig 2:**
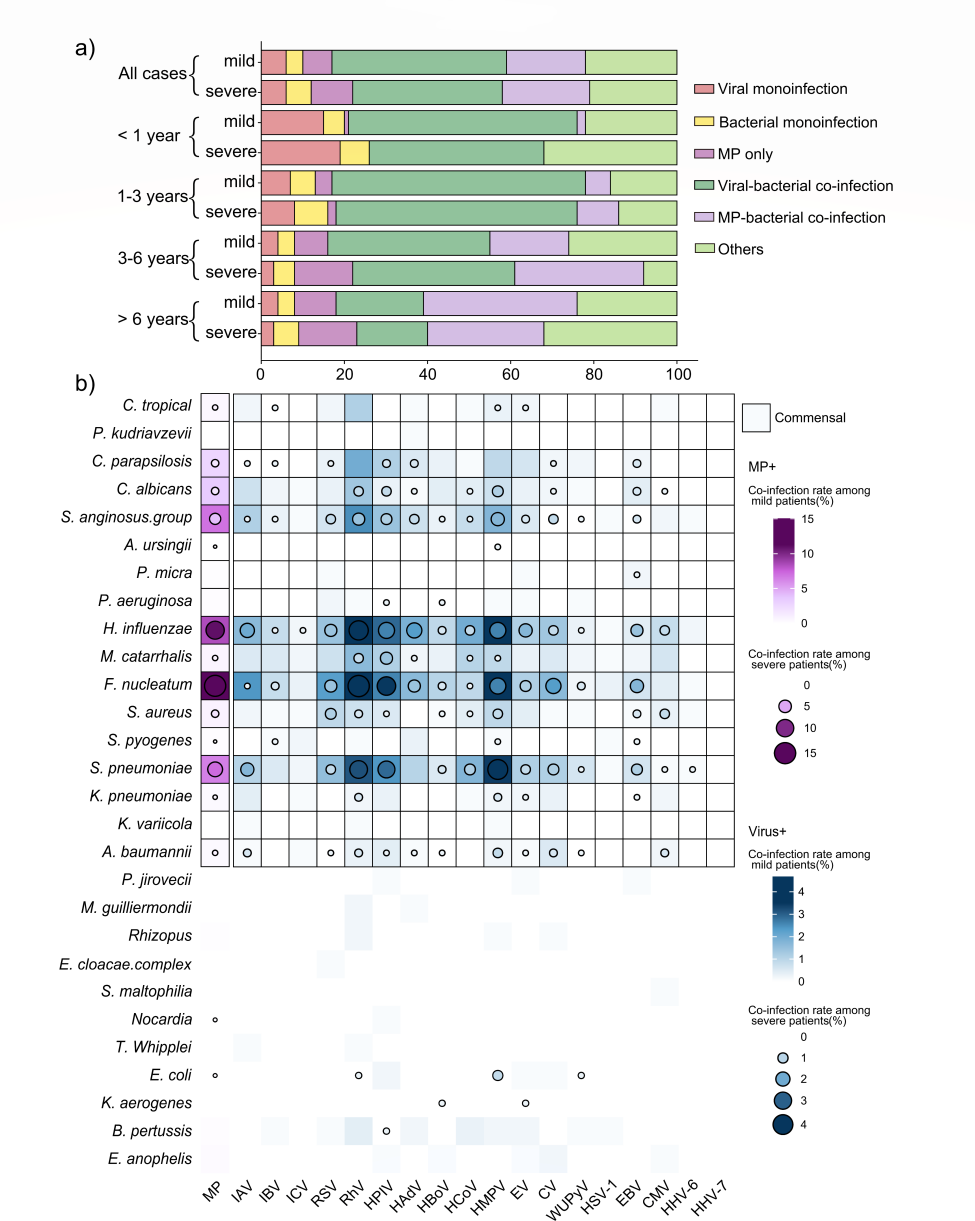
Co-infection patterns of MP and respiratory viruses in phase III (June to December 2023). (**a**) The proportion of children with viral mono-infection, bacterial mono-infection, MP mono-infection, or co-infections of viruses with bacteria, or MP with bacteria across different ages (in mild and severe groups). (**b**) Heatmap showing the rates of MP and viruses co-infected with respiratory bacteria or fungi. The color in the grid indicates the co-infection rate of respiratory pathogens among mild patients, while the color in the circle represents the co-infection rate among severe patients. Darker colors signify a higher co-infection rate between the pair of pathogens. Co-infection rate is defined as the number of samples that are positive for both pathogens A and B, divided by the total number of samples from patients with mild or severe symptoms, respectively.

In phase III, analysis of tNGS results from 1,308 throat swab samples revealed a total of 48 types of respiratory pathogens, including 19 viruses, 28 bacteria and fungi, and MP. Viral, bacterial, or MP mono-infections accounted for a small proportion, while the majority of pediatric patients had co-infections of multiple pathogens, with virus-bacteria/fungi co-infections accounting for 39.1% (511/1,308), MP-bacteria/fungi co-infections for 18.9% (247/1,308), and other co-infections (including virus-virus co-infections, bacteria-bacteria co-infections, etc.) for 21.3% (278/1,308) (Fig. 2a).

The infection patterns varied across different age groups (<1 year old, 1–3 years old, 4–6 years old, and 7–14 years old). Viral infections are higher in younger groups (Virus only: 16.2%, 6.9%, 3.5%, 3.7%; Virus+bac: 51.3%, 60.6%, 39.0%, 19.6% in four age groups from young to old, respectively) (Fig. 2a).

The primary viruses causing respiratory infections in phase III include RhV, HMPV, HPIV, IAV, and RSV, etc. *F. nucleatum*, *H. influenzae*, and *S. pneumoniae* were the top bacteria co-infected with these respiratory viruses, particularly the combinations of RhV-*F. nucleatum* (4.66% [61/1,308]), RhV-*H. influenzae* (4.28% [56/1,308]), and HMPV-*S. pneumoniae* (7.3% [54/1,308]). Notably, HMPV-*S. pneumoniae*, HPIV-*F. nucleatum,* and HPIV-*S. pneumoniae* had higher frequencies in severe cases (Fig. 2b). We further analyzed the 12 most common pathogen co-infection combinations shown in Fig. 2b (including MP, RhV, HPIV, and HMPV, each paired with *H. influenzae*, *F. nucleatum*, or *S. pneumoniae*) to investigate their associations with disease severity. The covariates—age, sex, and the number of days from symptom onset to hospital admission—were adjusted for using multivariate logistic regression analysis. HMPV-*S. pneumoniae* (OR = 3.172, 95% CI: 1.257–8.009), HPIV-*F. nucleatum* (3.016, 1.051–8.653), and HPIV-*S. pneumoniae* (2.825, 1.007–7.927) were associated with increased disease severity (Fig. S3).

### MP co-infections with *F. nucleatum*, *H. influenzae*, and *S. pneumoniae* are more frequently observed in severe cases

In contrast to viral infections, the proportion of MP infections increased with age (MP only: 0.9%, 4.0%, 9.3%, 11.3%; MP+bac co-infections: 1.7%, 6.6%, 22.1%, 34.1% in four age groups from young to old, respectively). Among all cases in phase III, the proportion of MP infections and MP-bacteria co-infections in severe patients is higher than that in mild patients (MP only: 9.7% [32/331] > 6.8% [64/937], MP+bac: 21.5% [71/331] >18.8% [176/937]) (Fig. 2a).

The most commonly detected bacteria co-infected with MP were *F. nucleatum*, *H. influenzae*, and *S. pneumoniae*, with higher frequencies observed in severe patients, accounting for 15.1% (52/345), 11.0% (38/345), and 7.3% (25/345), respectively (Fig. 2b). Among these, MP*-H. influenzae* was associated with severe ARI (OR = 1.797, 95% CI: 1.103–2.929) (Fig. S3A).

### MP abundances are positively correlated with EOS, IL-6, and IL-4, while negatively correlated with IL-2 and IgG

**Fig 3:**
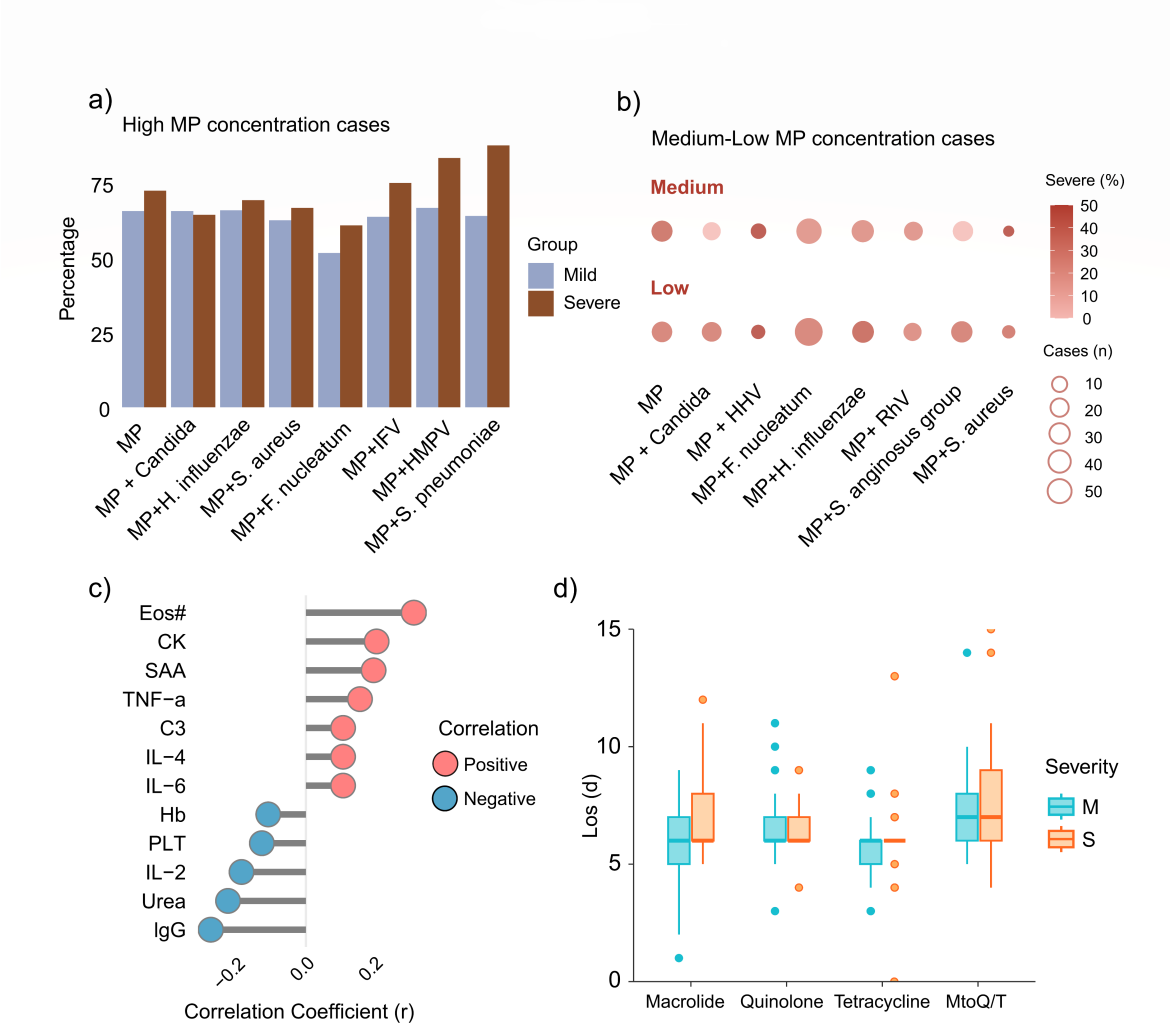
MP concentration correlates with immune indicators and influences severity. (**a**) Bar chart displays the proportion of cases with high MP concentration among mild or severe patients with different coinfections. (**b**) Co-detection of MP with various pathogens in medium and low MP concentration cases. Bubble size represents the number of co-infected cases, and color represents the percentage of severe cases among each condition. (**c**) Lollipop chart illustrating the correlation between Mycoplasma pneumoniae concentrations and clinical indicators in children with MP mono-infection. Negative correlations are indicated in blue, and positive correlations in red. (**d**) Boxplot displaying hospital length of stay (LOS) for children with MP infections across different treatment groups. The four medication regimens include macrolides (azithromycin), quinolones (levofloxacin), tetracyclines (tetracycline and minocycline), and M to Q/T treatment (referring to switching from macrolide treatment within less than 3 days to alternative drugs). Eos#, eosinophil count; CK, creatine kinase; SAA, serum amyloid A; TNF-α, tumor necrosis factor-alpha; C3, complement component C3; IL-4, interleukin-4; IL-6, interleukin-6; Hb, hemoglobin; PLT, platelet count; IL-2, interleukin-2; IgG, immunoglobulin G.

tNGS simultaneously reports the pathogens and their estimated concentrations. Patients with high MP loads mostly took a higher proportion in the severe group compared to the mild group (Fig. 3a). In patients with medium or low MP concentrations, certain co-infections were associated with a higher proportion of severe disease, such as MP-Candida (29.4%), HHV (50.0%), *F. nucleatum* (29.3%), and *H. influenzae* (40.0%) (Fig. 3b).

Further investigation indicated that peripheral eosinophil count (Eos#) showed the highest correlation with MP abundance. Conversely, hemoglobin concentration (Hb) and platelet count (PLT) were negatively correlated with MP loads. Inflammatory cytokines, tumor necrosis factor-alpha (TNF-α), interleukin-4 (IL-4), and interleukin-6 (IL-6), as well as complement C3, serum amyloid A (SAA), and creatine kinase (CK), were positively correlated with MP loads. Notably, interleukin-2 (IL-2) and immunoglobulin G (IgG) were negatively correlated with MP (Fig. 3c).

Our tNGS detection panel detected the A2063G mutation in the 23S rRNA of MP. For cases with A2063G^mut^ MP, antimicrobial treatments were adjusted to tetracyclines or fluoroquinolones. Similar to the first-line drug azithromycin treatment group, the median hospital stay for these alterations was 6 days. For children who had no obvious response to azithromycin treatment for 3 days, treatment was timely switched to tetracycline or fluoroquinolone, resulting in a hospital stay of 7 (6, 8) days (Fig. 3d).

### Elevated monocytes and neutrophils are CBC features of viral-bacterial co-infections

**Fig 4:**
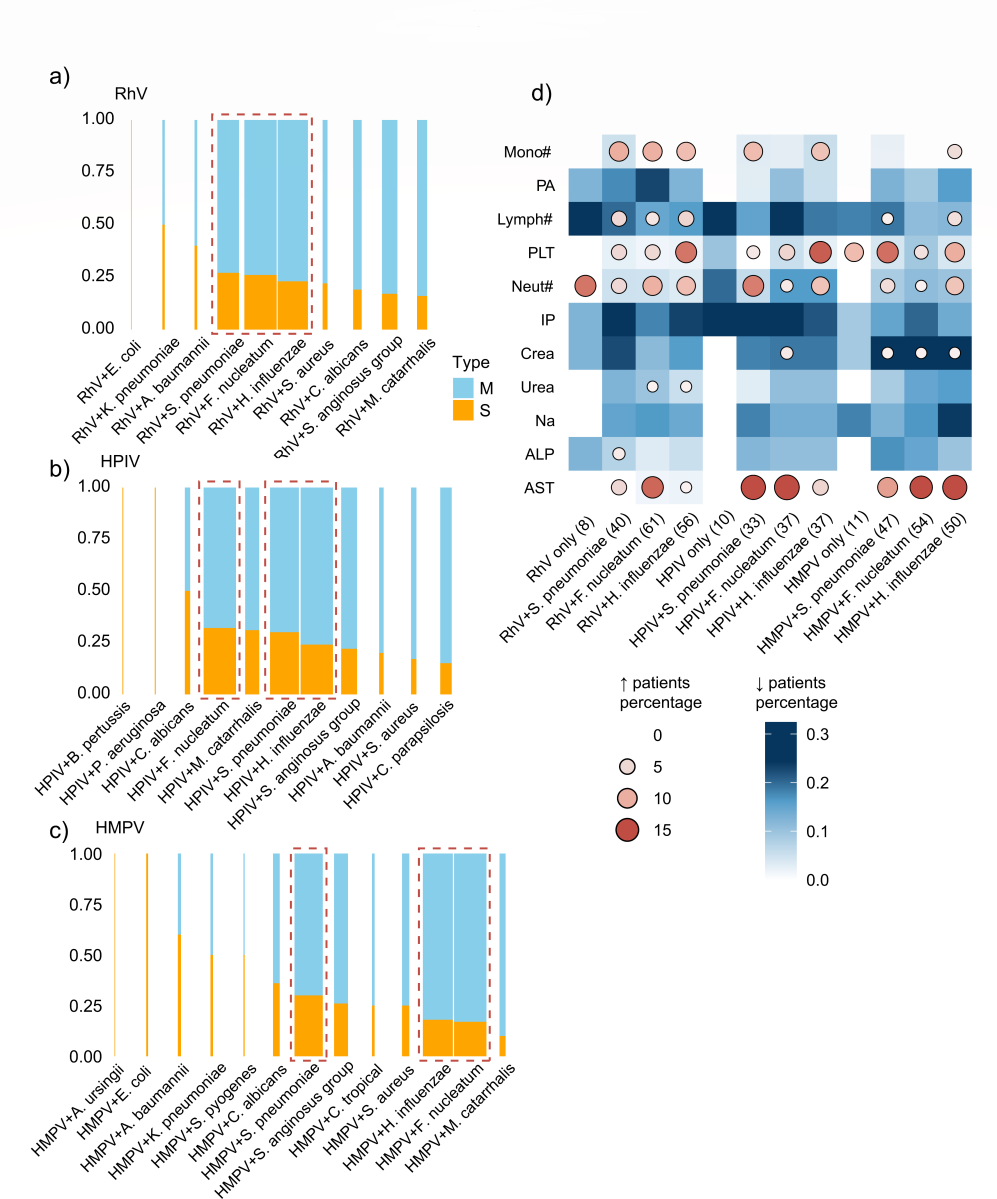
Blood indicators of RhV, HPIV, and HMPV co-infections with bacteria during phase III. (**a–c**) Weighted stacked bar chart showing the distribution of severe cases among RhV (**a**), HPIV (**b**), and HMPV (**c**) co-infections. Bar width represents the number of cases co-infected with the corresponding virus and bacteria. The orange and blue sections represent the proportion of severe and mild cases, respectively. (**d**) Abnormal values of clinical indicators for patients in different conditions of respiratory virus and viral-bacterial co-infections (including RhV, HPIV, HMPV, *F. nucleatum*, *S. pneumoniae*, and *H. influenzae*). Red in the circles indicates the proportion of children with values above the normal reference range; blue in the squares indicates the proportion of children with values below the normal range. Mono#, monocyte count; PA, prealbumin; Lymph#, lymphocyte count; PLT, platelet count; Neut#, neutrophil count; IP, inorganic phosphorus; Crea, creatinine; Na, sodium; ALP, alkaline phosphatase; AST, aspartate aminotransferase.

During phase III, a total of 316 cases involved co-infections of RhV, HPIV, or HMPV with bacteria. The most common co-detected bacteria were *F. nucleatum*, *S. pneumoniae*, and *H. influenzae*, and these co-infections showed a high number of severe cases (Fig. 2b and 4a through c). The proportion of severe cases of RhV-*F. nucleatum*, RhV-*S. pneumoniae*, and RhV-*H. influenzae* was 26.2% (16/61), 30.0% (12/40), and 23.2% (13/56), respectively. For HPIV co-infections, the proportion of severe cases was 32.4% (12/37) with *F. nucleatum*, 33.3% (11/33) with *S. pneumoniae*, and 21.6% (8/37) with *H. influenzae*. For HMPV co-infections, the proportion was 16.7% (9/54) with *F. nucleatum*, 29.8% (14/47) with *S. pneumoniae*, and 16.0% (8/50) with *H. influenzae*.

Children with viral-bacterial co-infections exhibited significantly different hematological and biochemical parameters compared to single viral infections (Fig. 4d). Specifically, the proportion of patients with increased monocytes and neutrophils was higher in groups of RhV, HPIV, or HMPV co-infected with *F. nucleatum*, *S. pneumoniae*, or *H. influenzae* compared to viral mono-infection groups. Over 12.5% of patients with RhV-*H. influenzae*, HPIV-*H. influenzae*, or HMPV-*S. pneumoniae* co-infections had an elevated platelet count. Lymphopenia is commonly seen after viral infections, but a few cases of RhV co-infections with *F. nucleatum*, *S. pneumoniae*, or *H. influenzae*, as well as HMPV co-infections with *S. pneumoniae* or *H. influenzae*, presented increased lymphocyte numbers.

### Subnormal serum prealbumin, creatinine, and urea levels are indicators of homeostasis disruption in viral-bacterial co-infections

Serum aspartate aminotransferase (AST) had no obvious change in cases with viral mono-infections but was elevated in patients with co-infections of RhV-*F. nucleatum*, HPIV-*S. pneumoniae*, HPIV-*F. nucleatum*, HMPV-*F. nucleatum*, and HMPV-*H. influenzae*. Compared to viral mono-infections, more patients exhibited decreased serum prealbumin (PA) in viral-bacterial co-infection groups, particularly in RhV-*F. nucleatum* (23.0% [14/61]), RhV-*S. pneumoniae* (17.5% [7/40]), and HMPV-*H. influenzae* (16.0% [8/50]) infections. Serum creatinine (Crea), urea, and alkaline phosphatase (ALP) showed similar decreases in viral-bacterial co-infections. Also, many patients had decreased serum inorganic phosphorus (IP) and sodium (Na) levels, especially in HPIV-related infections (each group >13.5%), which may be associated with electrolyte disorder (Fig. 4d).

## DISCUSSION

This study compiled data from pediatric in-patients with respiratory infections in Wuhan from June 2022 to December 2023, including case information, tNGS results, and clinical laboratory data. Our analyses investigated the prevalence trends and co-infection patterns of respiratory pathogens in three phases. We identified the dominant pathogens and co-infection combinations and further explored their correlations with disease severity and clinical characteristics.

In 2023, mainland China faced multiple waves of various respiratory pathogens. Viral infections predominate in infants (<1 year old) and toddlers (1–3 years old). We found that different respiratory viruses emerged in a staggered pattern: IAV peaked in March, RSV in April, RhV in May, and HPIV in June 2023. From July to December, HMPV and RhV had small peaks and remained detectable. The mechanisms underlying this “niche-occupied-like” effect may involve direct interactions of viral gene products, indirect interactions through alterations in the host environment, and immunological crosstalk (12–14). In pediatric populations, physiological consumption and immune influence from previous infections could increase susceptibility to subsequent pathogens, highlighting the great necessity for vigilance and prevention of respiratory viruses rebound after a pandemic.

Notably, in our study, the peak detection of CMV in children coincided with the SARS-CoV-2 Omicron wave, and CMV or EBV co-detection was identified in 103 pediatric cases infected with RSV, RhV, or other respiratory viruses. Previous studies have shown that CMV/EBV rebound accompanied by primary infection is associated with worse clinical condition and prolonged hospital stays (15, 16), highlighting the importance of monitoring these viral co-detection events for potential disease exacerbation and long-term sequelae.

Respiratory viral infections increase the susceptibility to secondary infections and overgrowth of commensals, potentially leading to invasive infections, such as bacteremia and purulent meningitis (17–19). In our study, single-pathogen respiratory infections accounted for 18.4%, while viral-bacterial and MP-bacterial co-infections accounted for 56.3%. In phase III, co-detection of MP, RhV, HPIV, and HMPV with *H. influenzae*, *F. nucleatum*, and *S. pneumoniae* was most prevalent among pediatric patients. Severe cases more frequently involved HMPV co-infection with *S. pneumoniae*, HPIV co-infections with *F. nucleatum* or *S. pneumoniae*, and MP co-infections with *H. influenzae. F. nucleatum* has been increasingly recognized for its role in precipitating or exacerbating respiratory infections, inducing cases of pneumonia (20). In our study, the positive detection rate of *F. nucleatum* by tNGS was about 38.9% among in-patients, mostly co-detected with viruses or MP, indicating that preceding infections may promote *F. nucleatum* colonization. Further monitoring is required to understand its long-term consequences.

Severe infections correlate with the likelihood of severe or extreme thrombocytosis (21). Our study found that over 12.5% of patients with co-infections of RhV-*H. influenzae*, HPIV-*H. influenzae*, and HMPV-*S. pneumoniae* presented higher platelet counts than those with single viral infections. Key indicators of children’s physical condition and nutrition significantly changed: in viral-bacterial co-infections, especially RhV-*F. nucleatum*, RhV-*S. pneumoniae,* and HMPV*-H. influenzae*, levels of prealbumin (PA), creatinine (Crea), and urea (Urea) decreased. Reports indicate that malnutrition and related metabolic changes are important causes of acute symptoms and long-term health issues, and early nutritional risk assessment and support can improve clinical outcomes in severe infection patients (22–24). Our analyses indicated that individuals with weaker physical quality and poor nutritional status may be more susceptible to multiple infections; therefore, attention should be paid to secondary infections in nutritionally at-risk children, emphasizing the need for robust nutritional management and metabolic intervention strategies.

Our statistical analysis revealed that MP infections increased in preschoolers (4–6 years) and school-aged children (>6 years). Over 80% of MP cases among hospitalized children had co-infections, with *F. nucleatum, S. pneumoniae*, rhinovirus, herpesvirus, influenza virus, *C. albicans*, etc. Patients with high MP concentrations account for a larger proportion in severe cases, while in cases of low MP concentration, co-infection with herpesvirus or Candida may aggravate the defense consumption or immune imbalance (25), influencing severity. Not only do these findings suggest that tNGS-reported MP concentrations are prognostic for disease severity, but they also highlight the need for timely detection of co-infected pathogens to enhance the quality and efficiency of MP diagnosis and treatment.

Further analyses suggest that elevated MP levels were linked to decreased IL-2 and IgG, potentially reflecting an immunosuppressed state in some children with severe ARIs. In contrast, high MP levels had a positive correlation with SAA, TNF-α, IL-6, and IL-4. We also found the highest correlation between eosinophil count and MP loads, with children exhibiting increased EOS having more wheezing, dyspnea, cough symptoms, and elevated IgE levels. In line with the previous reports on MP promoting oxidative stress and raising risk for secondary allergies or asthma (26, 27), our results further highlight the need to monitor MP patients with elevated Th2 cytokines and EOS.

Resistance to macrolides in MP mainly involves mutations in the 23S rRNA gene at positions A2063G, A2064G, or C2617A (28, 29). Our tNGS results revealed macrolide-resistant mutations, all of which were A2063G. Patients infected with A2063G^mut^ MP were treated with doxycycline/minocycline or levofloxacin. The alternative treatment group had a median hospital stay similar to that of the azithromycin treatment group, which underscores the clinical value of tNGS in guiding antibiotic selection and reducing the risk of developing resistance.

In summary, our integrated analyses of tNGS results and clinical data revealed distinct characteristics for viruses or MP along with their co-infections, offering valuable insights into clinical diagnosis and surveillance. Early adoption of tNGS testing ensured favorable outcomes for all pediatric patients in this study, with no critical cases or adverse outcomes, robustly endorsing the reliability and utility of broad-spectrum tNGS in the management of ARIs.

## Data Availability

The tNGS data reported in this study have been deposited in the OMIX, China National Center for Bioinformation, under accession code OMIX011224 and are publicly accessible at https://ngdc.cncb.ac.cn/omix. Data are available from the corresponding author upon request.

## SUPPLEMENTAL MATERIAL

Supplemental material spectrum.02275-25-s0001.pdf Table S1; Fig. S1 to S3.

ASM does not own the copyrights to Supplemental Material that may be linked to, or accessed through, an article. The authors have granted ASM a non-exclusive, world-wide license to publish the Supplemental Material files. Please contact the corresponding author directly for reuse.
