# Supplementary material for: Pathogen landscape and synergistic interactions in pediatric ARIs: implications for broad-spectrum surveillance via targeted NGS
Source: Microbiol Spectr. 2025 Dec 30;14(2):e02275-25. doi: 10.1128/spectrum.02275-25 (PMC12889064; doi:10.1128/spectrum.02275-25)
Supplement: Supplemental material — Table S1; Fig. S1 to S3. [file spectrum.02275-25-s0001.pdf]

Supplementary Material to

Pathogen landscape and synergistic interactions in pediatric ARIs: implications for broad-spectrum surveillance via targeted NGS

Supplementary Table S1. The baseline characteristics of enrolled paediatric in-patients, 2022.6-2023.12.

Supplementary Figure S1. Monthly trends in the positive rates of respiratory viruses among paediatric patients from June 2022 to December 2023.

Supplementary Figure S2. Monthly trends in the positive rates of M. pneumoniae, respiratory bacteria, and fungi in paediatric patients from June 2022 to December 2023.

Supplementary Figure S3. Increased incidence of severe ARI was associated with certain pathogen combinations.

Supplementary Table S1. The baseline characteristics of enrolled paediatric in-patients, 2022.6-2023.12.

	Total (N=2044)	Phase I (n=211)	Phase II (n=525)	Phase III (n=1308)	*p*- value
Sex					
Female	918 (44.9%)	82 (38.9%)	245 (46.7%)	591 (45.2%)	0.149
Male	1126 (55.1%)	129 (61.1%)	280 (53.3%)	717 (54.8%)	
Age group					
0-6 months	143 (7.0%)	19 (9.0%)	56 (10.7%)	68 (5.2%)	
7-11months	104 (5.1%)	11(5.2%)	31 (5.9%)	62 (4.7%)	<0.001[a]
1-3 years	723 (35.4%)	97 (46.0%)	234 (44.6%)	392 (30.0%)	
4-6 years	554 (27.1%)	43 (20.4%)	126 (24.0%)	385 (29.4%)	
>7 years	520 (25.4%)	41 (19.4%)	78 (14.9%)	401 (30.7%)	
Diagnosis					
Pneumonia	1020 (50.0%)	60 (28.4%)	214 (40.8%)	746 (57.0%)	<0.001[a]
Non-pneumonia	1024 (50.0%)	151 (71.6%)	311 (59.2%)	562 (43.0%)	
tNGS detected pathogens					
Yes	1943 (95.1%)	193 (91.5%)	485 (92.4%)	1265 (96.7%)	0.197
No	101 (4.9%)	18 (8.5%)	40 (7.6%)	43 (3.3%)	

Data are n (%). Phase I: June to December 2022; Phase II: January to May 2023; Phase III: June to December 2023. [a] Statistically significant changes were found (*p* value <.05 based on chi-square test).

Supplementary Figure S1. Monthly trends in the positive rates of respiratory viruses among paediatric patients from June 2022 to December 2023.

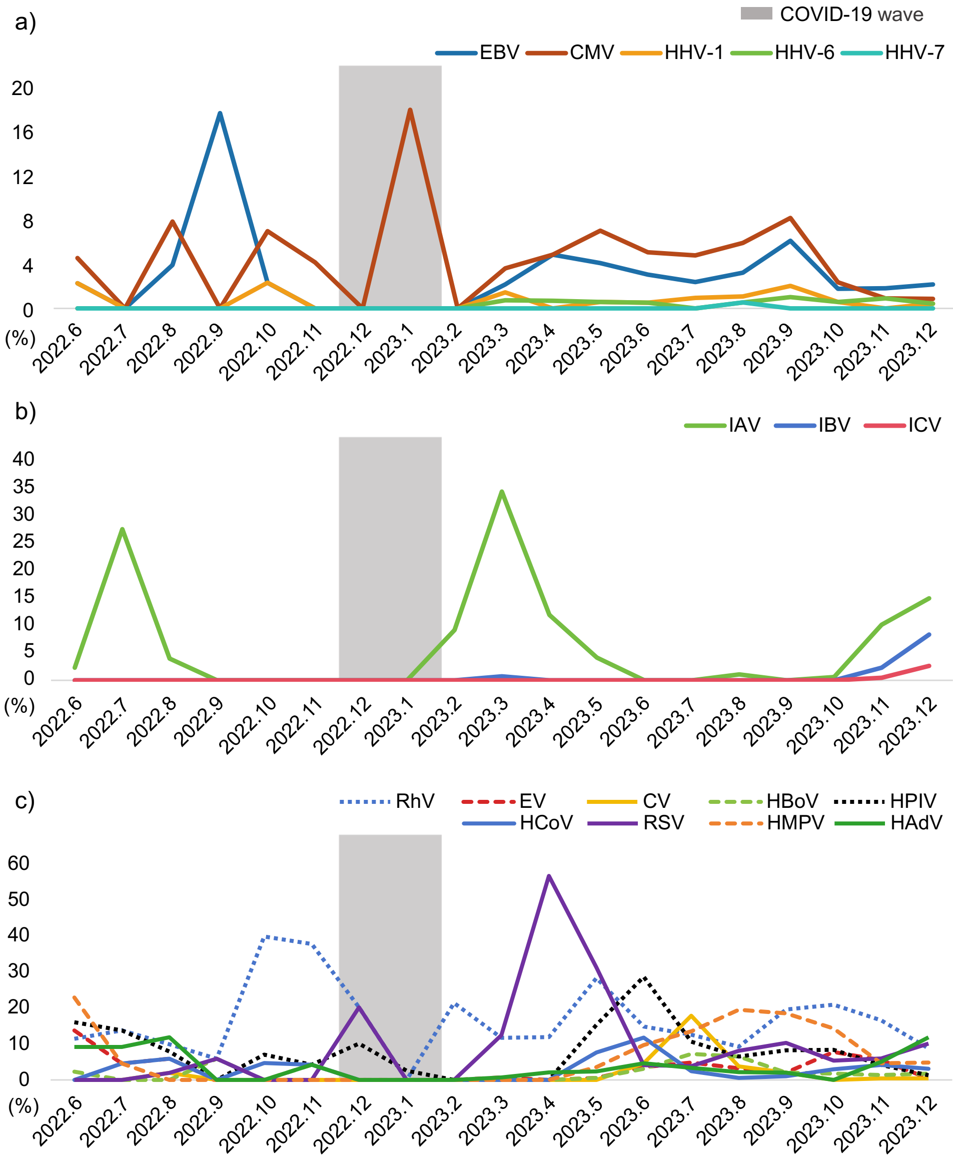

(A-C) The tNGS-detected positive rates of different subtypes of herpesviruses **(A)**, influenza viruses **(B)**, and other common respiratory viruses that can cause the common cold **(C)** in children hospitalized with ARIs from June 2022 to December 2023, indicated with different colored lines. The positive rate is calculated as the number of positive cases divided by the total number of samples tested each month. The grey-shaded areas represent the period of COVID-19 Omicron outbreak in Wuhan.

Supplementary Figure S2. Monthly trends in the positive rates of M. pneumoniae, respiratory bacteria, and fungi in paediatric patients from June 2022 to December 2023.

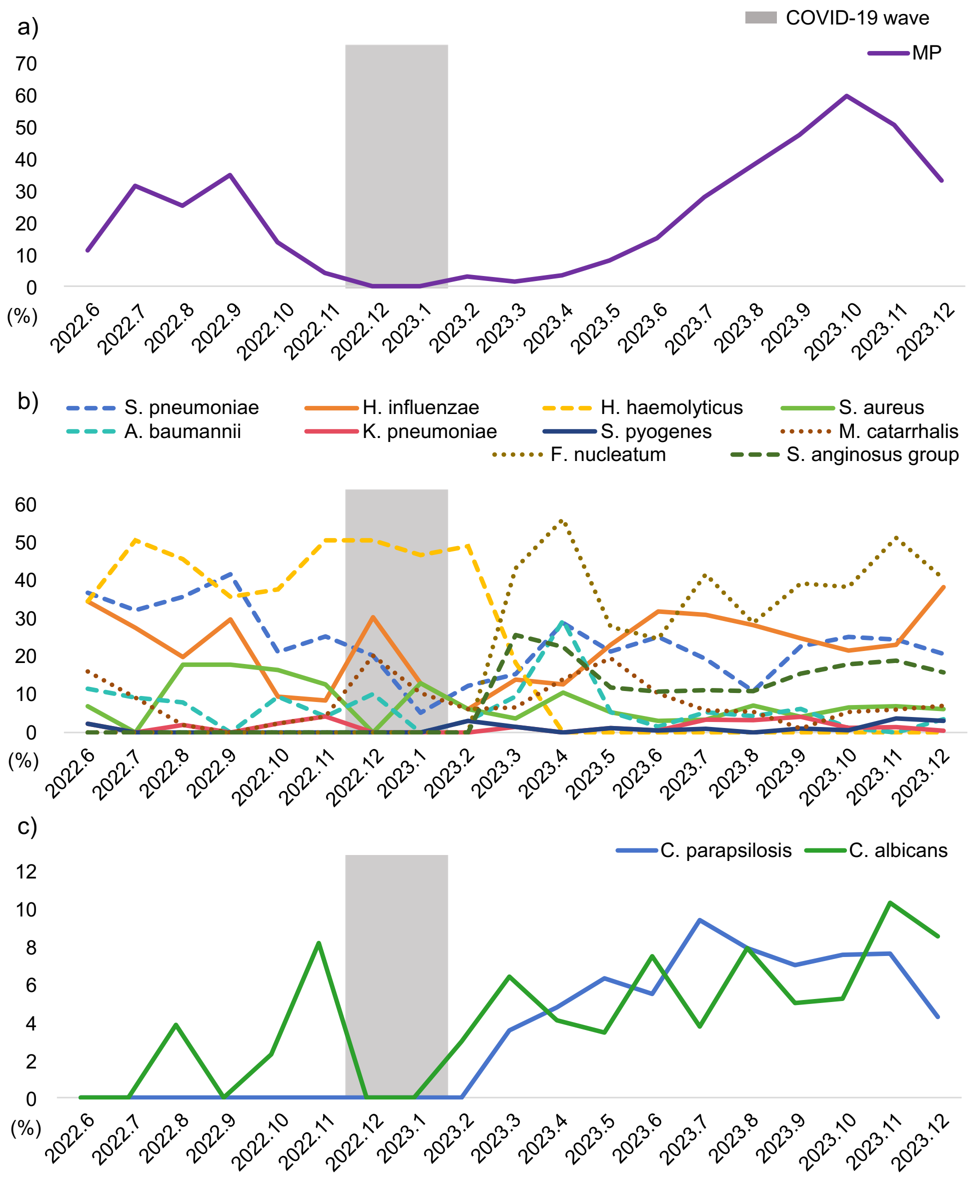

(A-C) The tNGS-detected positive rates of Mycoplasma pneumoniae **(A)**, bacteria associated with respiratory infections **(B)**, and the fungi mainly associated with respiratory infection **(C)** in children hospitalized with ARI from June 2022 to December 2023, indicated with different colored lines. The positive rate is calculated as the number of positive cases divided by the total number of tested samples each month. The grey shaded areas represent the period of COVID-19 Omicron outbreak in Wuhan. MP, Mycoplasma pneumoniae.

Supplementary Figure S3. Increased incidence of severe ARIs was associated with certain pathogen combinations.

	p_value	adjusted OR (95%CI)
S.pneumoniae_HMPV	0.007 *	3.172(1.257−8.009)
F.nucleatum_HPIV	0.024 #	3.016(1.051−8.653)
S.pneumoniae_HPIV	0.034 #	2.825(1.007−7.927)
H.influenzae_MP	0.009 *	1.797(1.103−2.929)
F.nucleatum_RhV	0.320	1.561(0.606−4.018)
S.pneumoniae_RhV	0.417	1.430(0.549−3.724)
F.nucleatum_MP	0.234	1.270(0.814−1.979)
days_onset_admission	0.004 *	1.044(1.013−1.077)
age	0.754	1.008(0.957−1.063)
H.influenzae_RhV	0.957	0.977(0.412−2.314)
sex_Female	0.830	0.967(0.711−1.315)
S.pneumoniae_MP	0.527	0.841(0.453−1.564)
H.influenzae_HMPV	0.256	0.600(0.219−1.641)
H.influenzae_HPIV	0.085	0.406(0.130−1.271)
F.nucleatum_HMPV	0.024 #	0.331(0.116−0.949)

FDR < .1
* FDR < .05

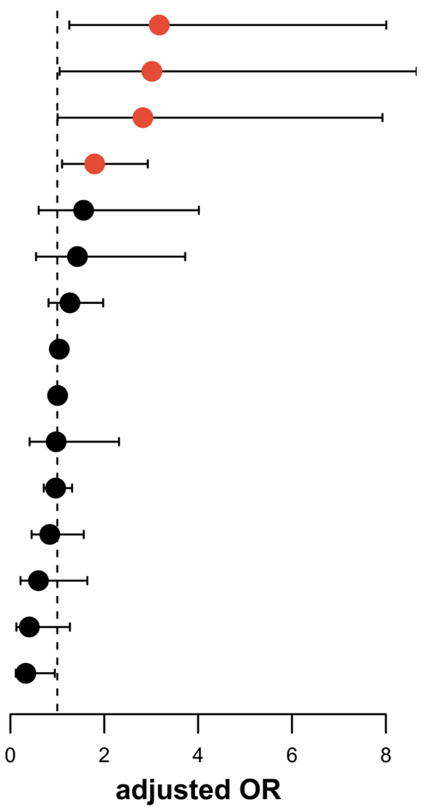

The association between co-infections with severe ARI development was explored by logistic regression analysis. Forest plot displays the odds ratio (OR) and 95% confidence intervals (CI). Statistically significant results are colored red (for OR>1).
